# Non-invasive diagnosis of acute rejection in renal transplant patients using mass spectrometry of urine samples - a multicentre phase 3 diagnostic accuracy study

**DOI:** 10.1186/s12882-015-0146-x

**Published:** 2015-09-15

**Authors:** Antonia Zapf, Wilfried Gwinner, Annika Karch, Jochen Metzger, Hermann Haller, Armin Koch

**Affiliations:** Institute for Medical Statistics, University Medical Center Göttingen, Humboldtallee 32, 37073 Göttingen, Germany; Department of Nephrology, Medical School Hannover, Carl-Neuberg-Str. 1, 30625 Hannover, Germany; Institute for Biostatistics, Medical School Hannover, Carl-Neuberg-Str. 1, 30625 Hannover, Germany; Mosaiques Diagnostics and Therapeutics, Rotenburger Str. 20, 30659 Hannover, Germany

**Keywords:** Kidney transplantation, Acute rejection, Allograft biopsy, Urine analysis, Mass spectrometry, Urinary peptides, Diagnosis, Diagnostic study, Sensitivity, Specificity

## Abstract

**Background:**

Reliable and timely detection of acute rejection in renal transplant patients is important to preserve the allograft function and to prevent premature allograft failure. The current gold standard for the rejection diagnosis is an allograft biopsy which is usually performed upon an unexplained decline in allograft function. Because of the invasiveness of the biopsy, non-invasive tests have been suggested to diagnose acute rejection including mass spectrometry analysis of urine samples.

**Design and methods:**

The aim of this study is to examine the diagnostic accuracy of mass spectrometry analysis in urine for the diagnosis of acute rejections using the biopsy as gold-standard. The study is an ongoing prospective, single-arm, multicentre, phase 3 diagnostic accuracy study. It started in October 2011 and will be concluded in December 2015. Patient within the first year after transplantation who are scheduled for a biopsy to clarify unexplained impairment of the allograft are consecutively recruited into the study. The overall sample size (n = 600) was calculated to demonstrate a sensitivity of 83 % and a specificity of 70 % for a one-sided type one error of 2.5 % and a power of 80 % per hypothesis. Biopsy evaluation and mass spectrometry analysis of urine samples (obtained immediately before biopsy) are performed independently by different readers without knowledge from the respective other assessment. The follow-up observation period is 6 months. For the primary analysis, the lower limits of the two-sided 95 % Wald confidence intervals for sensitivity and specificity will be compared with the pre-specified thresholds (83 % for sensitivity and 70 % for specificity). In secondary analyses the predictive values, the diagnostic measures in subgroups, and the clinical course will be assessed.

**Discussion:**

Previous phase 2 diagnostic accuracy studies (in small selected study populations) provided sufficient evidence to suggest mass spectrometry on urine samples as a promising approach to detect acute rejections. This study determines the diagnostic performance of the test in the routine setting of post-transplant patient care, compared to the biopsy-based rejection diagnosis. The next step would be a randomized trial to compare the two diagnostic strategies (including the urine test or not) in relation to patient relevant endpoints.

**Trial registration:**

NCT01315067; March 14, 2011

**Electronic supplementary material:**

The online version of this article (doi:10.1186/s12882-015-0146-x) contains supplementary material, which is available to authorized users.

## Background

Acute rejection is an important factor that determines the long-term function and survival of the renal allograft. Therefore, timely detection and appropriate treatment of acute rejections is an important objective in the post-transplant care. Approximately 15-30 % of the transplanted patients suffer from one or multiple episodes of acute rejection which occur mainly in the first year of transplantation [1]. The most common type of acute rejection − with more than 90 % − is the T-cell mediated tubulointerstitial rejection (Ia and Ib according to BANFF classification). Acute T-cell mediated vascular rejection and acute humoral rejection are less frequent in the first transplant year [2, 3].

The detection of acute rejection in an early stage is challenging [4]. The current standard in the allograft surveillance relies on regular monitoring for increases in serum creatinine or a decrease in creatinine clearance which then triggers subsequent biopsy. This implies that acute rejections are diagnosed at a stage where the morphological damage already has led to a relevant functional impairment of the graft. Because of this problem some transplant centres perform protocol biopsies as part of the post-transplant care which can detect acute rejections at an early subclinical stage before functional impairment has occurred. However, biopsies are invasive and even with frequent regular protocol biopsies rejection episodes may be missed.

Therefore, many attempts have been made to develop non-invasive tests in blood or urine which are able to detect acute rejection. Most of these approaches used single markers or combinations of a few markers. Mostly due to lacking specificity and sensitivity of these markers, none of these tests are established in the clinical routine and post-transplant care of the patients [4]. An alternative approach, which takes in account the heterogeneity of the rejection process, is the use of multiple markers as detected by mass spectrometry in urinary samples.

A few diagnostic phase 1 and phase 2 studies with such non-invasive blood and urine tests (according to [5, 6]) demonstrated promising results regarding sensitivity and specificity for the diagnosis of rejection [7–12]. However, until now the validation of these approaches in a representative study population and in the clinical routine of the post-transplant is still missing thus prohibiting a realistic estimation of sensitivity and specificity of these tests.

Beside the small sample sizes in these studies there are some further limitations. The histological scoring among the different studies is not uniformly conducted according to the BANFF classification [7, 9]. Partially, histological confirmation of the absence of rejection in the test-negative patients is missing (leading to the so called differential verification bias). Another important limitation is that, due to the small sample sizes, effects of potential confounders, like infectious conditions or different immunosuppressive regimens, are unknown.

Extending our proof-of concept study on 26 patients with acute tubulointerstitial rejection [10], we started out to refine and standardize the sample processing and analysis [13]. The resulting peptide pattern was validated in a cohort of 25 patients with and 43 patients without rejection. The AUC value was calculated to be 0.89 (95 % CI: 0.80 – 0.95). In a replication with a blinded test set of 17 independent patients, 6 of 7 patients with acute rejection were correctly classified as having rejection and 10 of 11 samples were correctly classified as negative for rejection (AUC: 0.83; 95 % CI: 0.57-0.96).

Moreover, we were able to identify the sequences of four peptides of the rejection pattern and found that three are fragments of the collagen type I alpha(I) (Col1A1) whereas the other is collagen type I alpha(III) derived. Similar results with changes of Col1A1 fragments in acute renal allograft rejection have been reported very recently [12]. In our peptide pattern, two of the Col1A1 fragments were less abundant in the rejection samples and one was increased. Aligning these fragments to other, commonly found Col1A1 peptides in the urine samples, we found that the increased peptide (ID-114633) possess a PGP motif at the c-terminal end which is indicative of increased matrix degradation by MMP-8 and MMP-9. Double-stains of the biopsies for MMP-8 and for an endothelial cell marker revealed confinement of MMP-8 to polymorphonuclear leukocytes located within pertubular capillaries and sparsely, in the interstitial infiltrates and glomerular capillaries of biopsies with rejection [13].

Given all these facts, sufficient evidence exists to suggest mass spectrometry on urine samples as a promising approach to detect acute rejection. However, proof is lacking that this test provides good diagnostic performance in the routine setting of the post-transplant patient care, compared to the biopsy-based rejection diagnosis.

Therefore, the aim of this multicentre prospective phase 3 diagnostic accuracy study is to investigate if the mass spectrometry (MS) test developed by Metzger et al. [13], is useful in a representative setting of the clinical routine post-transplant care. Meticulous collection of clinical data including potential confounders and use of rigorous histological standards (BANFF classification, repeat central pathology reading of biopsies) in this trial will maximize the interpretation of the obtained mass spectrometric results. At the end, reliable estimators of the diagnostic accuracy (sensitivity, specificity, predictive value, limits of the test) will be derived from the trial.

## Methods/Design

### Study aims and objectives

The aim of the study is to prove that the MS test has good diagnostic performance compared to the allograft biopsy to detect acute tubulointerstitial rejection in renal-transplant patients who undergo a biopsy for unexplained renal dysfunction.

The primary objective of the trial is to show that mass spectrometry is not relevantly inferior to the gold-standard allograft biopsy regarding sensitivity and specificity.

The secondary objectives are to evaluate the predictive values of the MS test and to investigate the diagnostic accuracy of the MS test in subgroups, namely in patients with different histological severity of the rejection, with different severity of functional impairment, with kidney versus a combined kidney/pancreas transplant, with potential confounders present (e.g. urinary tract infection, concurrent CMV infection, polyomavirus nephropathy), and in patients from different study centres.

### Study design

This study is a prospective multicentre phase 3 diagnostic accuracy study (according to [5, 6]) for the intra-individual comparison of the MS test and the allograft biopsy. The MS test is the index test, while the allograft biopsy is the gold standard. Biopsy and mass spectrometry will be performed independently by different readers without knowledge from the respective other assessment. Results of the MS test will be kept concealed until the end of the study. Accordingly, this is a non-interventional trial because the treatment is guided by the result of the allograft biopsy, as the current diagnostic standard. All treatments are permitted which are deemed necessary by the clinicians caring for the patients.

### Study setting and period

This study is carried out in 10 German university and non-university transplant centres: Hannover, Aachen, Jena, Erlangen, Freiburg, Essen, München, Köln, Berlin, Hannoversch Münden. It is expected that virtually all patients who are scheduled for an indicated biopsy agree to participate in the study because of the non-interventional nature of the trial. Drop-out rate will be negligible for primary analysis because once a patient has consented to participate in the study the essential data set is available for analysis.

Data collection was initiated in October 2011 and the conclusion of this study is scheduled for December 2015.

### Participants/eligibility criteria

The test should be applicable to all kidney or kidney/pancreas transplant patients in the post-transplant care which are scheduled for an indicated biopsy to verify clinical symptoms of an acute rejection. Therefore, inclusion/exclusion criteria are set out to the least limitations that are necessary to perform the study meaningfully, i.e. availability of the results of the comparator ‘renal allograft biopsy’. The inclusion of patients within the first year of transplantation is based on the fact that acute rejections are most frequent in this period. Thus, this population is suitable for estimation of sensitivity and specificity with high accuracy. Furthermore for this population the test would have the greatest relevance.

The inclusion criteria imply that female and male patients are likewise studied. As this study is carried out in the setting of adult renal allograft recipient post-transplant care, results will be applicable to all adult patients. Enrolment to the study is only allowed once for each patient, i.e. patients with repeated episodes of suspected acute rejection are not included a second time.

### Recruitment, procedures and follow-up

After identification of a patient with unexplained allograft function decline (within the first year after transplantation) and the clinical decision to perform a biopsy, informed consent is obtained from the patient to use an urine sample from the day of biopsy and the clinical data for the study.

The urine specimen is obtained immediately before the biopsy as a spot sample. Mass spectrometry is performed on this sample, along with the usual clinical routine determinations (protein concentration and sediment analysis).

The follow-up observation period after acquisition of the sample is 6 months. Main parameters to be used from the follow-up period are changes in renal allograft function (calculated GFR at 6 months after the biopsy in relation to the GFR before and at the time of biopsy) and further acute rejection episodes. For the assessment of allograft function 6 months after inclusion in the study, a regular out-patient visit will be scheduled for this time point. If the follow-up visit is impossible, all efforts will be made to obtain the necessary information from the primary care physician of the patient.

**Fig. 1:**
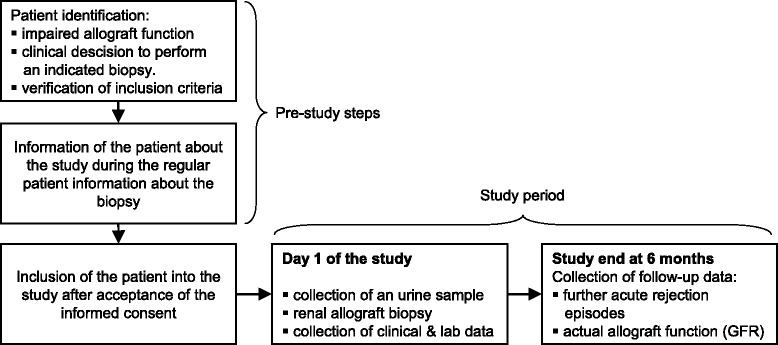
Trial flow (including pre-study steps)

For the illustration of the trial flow see Fig. 1.

### Index test

The index test uses CE-MS technology. Compared with other spectrometric and spectroscopic technologies, i.e., SELDI, NMR, the CE-MS technology offers a wider and more adjustable measurement range which is particularly suited for peptides and small proteins of approximately 9 to 180 amino acids in length. Thus, this technology is more sensitive for the detection of peptides in complex sample matrices (~1 fmol). It facilitates a higher degree of throughput (<1 h per sample) and automation (on-line coupling of CE and MS data acquisition via contact-close relays).

For the index test ‘acute rejection diagnosis by CE-MS’ neither internationally accepted pre-defined peptides for the rejection diagnosis, nor units, cut-offs or categories are available.

Classification of patient samples with the rejection pattern is expressed as membership probability and quantified by the Euclidean distance of the data point to the maximal margin of a separation hyperplane between cases and controls in a multidimensional space constructed by the peptide classifiers (by the use of Support Vector Machines, SVM). Membership probability therefore expresses the degree of similarity of a patients’ peptide profile to the one that was identified to be specific for the disease in the discovery phase on histomorphologically well-defined patients.

Test results of the samples will be classified into the categories ‘rejection’ and ‘no rejection’ by the pre-specified urinary peptide patterns and algorithms at the cut-off point of −0.25 based on the evaluation of CE-MS profiles of patients included in the proof-of-concept study.

### Standard of truth

The true disease state is assessed by biopsy according to the established BANFF criteria because this is the only accepted standard of truth for acute rejection diagnosis. This standard of truth is used throughout all relevant clinical trials and in the clinical routine to diagnose or to rule out acute rejection in renal transplant patients [14].

It is conceivable that the mass spectrometry test may detect acute rejections that are missed by biopsy because of the focal nature of the rejection in the allograft or because of potential biopsy sampling errors. Therefore, a sensitivity analysis will be performed secondarily for the cases with a positive mass spectrometry result and a negative biopsy, by using the clinical course and subsequently performed biopsies (if available) for an assessment of the probable true disease state at the time of first diagnosis.

### Clinical data

Clinical data include demographics of recipient and donor, immunologic parameters like HLA match and pre-formed antibodies, calculated GFR at the time of biopsy, any relevant diagnoses of the allograft like hydronephrosis or delayed graft function, any relevant factors at the time of biopsy which are potential confounders of the mass spectrometry test. These include CMV and polyomavirus infection, urinary tract infection, diabetes mellitus, hypertension, and the patient’s medication and immunosuppressive therapy.

### Outcome measures

The co-primary outcomes are sensitivity and specificity of the marker set for the diagnosis of acute rejection in the whole study population to determine the diagnostic accuracy of the marker set. These measures definitely determine whether the performance of the index test is sufficient to be used for the acute rejection diagnosis in comparison to the gold standard ‘renal allograft biopsy’.

Key secondary outcomes are *(i)* predictive values in terms of acute rejection in the whole study population and *(ii)* sensitivity, specificity and predictive values in subgroups. The subgroup analysis will help to determine the limits of the index test. This refers particularly to effects of confounding factors or conditions on the performance of the test and to the question which types and severity grades of acute rejection (in histological and functional terms) are detected with confidence.

### Statistical issues

#### Sample size

The primary objective of the trial is to show that mass spectrometry is not relevantly inferior to the gold-standard allograft biopsy. The null hypothesis of the study is that sensitivity and specificity of mass spectrometry are relevantly reduced compared to the gold standard biopsy. Thresholds are set to 83 % for sensitivity and to 70 % for specificity. Sample size calculation was done with nQuery 4.0, separately for sensitivity and specificity. The pre-test probability of acute rejection in kidney or kidney/pancreas transplanted patients with indication biopsy is expected to be 25 % (Hannover Transplant Centre Data; unpublished). The estimators of the test set of the previous phase II study are 91 % for sensitivity and 76 % for specificity. For both sample size calculations a one-sided χ^2^-test for single-proportion is used, assuming a one-sided type one error of 2.5 % and setting the power to 80 %. For a sensitivity of 83 % under the assumption of a true sensitivity of 91 %, 150 patients with acute rejection are needed, for a specificity of 70 % under the assumption of a true specificity of 76 %, 440 patients without acute rejection are needed. So, assuming the pre-test probability of 25 %, in sum a total sample size of 600 patients should be sufficient, where the patients with acute rejection (150 as 25 % of 600) determine the overall required number of patients.

No adjustment for multiplicity is necessary for testing sensitivity and specificity because both hypotheses have to be rejected, if the study should be evaluated as success. Loss to follow-up will not affect the analysis of primary and secondary outcomes because all necessary data for these calculations are collected at the time of urine sampling and renal biopsy on day 1 of the study.

#### Methods against bias

Biopsy and mass spectrometry will be performed independently by different readers. Readings must have reference to the medical history of the patients, but diligence will be exercised that knowledge of the results of the other test is withheld. For this reason, results of the experimental test will not be provided for patients and examiners that perform the gold standard test until the end of the study.

In addition to the local biopsy reading in each centre, biopsies will be re-evaluated centrally to confirm the interpretation of the biopsy. The index test is conducted according to a pre-assigned SOP (for details see Additional file 1).

Selection bias in the patient recruitment is negligible because the inclusion and exclusion criteria enable the study of a representative cohort by consecutively including all patients with an indicated biopsy who agree to participate in the study. The primary analysis will be confined to the indication biopsy and concomitant urine sample which were taken at day 1 of the study. Repeated urine collections from one patient will be not included into the primary analysis to avoid dependent data in a setting of independent observations from individual patients.

To assess the possible measurement error of the mass spectrometry, 10 % of the urine samples (randomly chosen) will be re-run including all sample preparation steps. In the secondary analysis, the results of these probe pairs will be used to evaluate the test reproducibility.

Impact of centre on the results will be examined in the statistical analysis.

#### Statistical analysis

For the primary analysis, point estimators and two-sided 95 % Wald confidence intervals for sensitivity and specificity will be calculated. The null hypothesis for sensitivity will be rejected if the lower bound of the corresponding confidence interval is above 83 %. The null hypothesis for specificity will be rejected if the lower bound of the corresponding confidence interval is above 70 %.

For the key secondary analyses, predictive values for all patients and sensitivity, specificity and predictive values for subgroups will be analysed descriptively. Subgroups are: patients with different BANFF grades of acute rejection; patients with different severity of acute rejection in terms of functional impairment at the time of rejection; patients with kidney allografts alone and patients with combined kidney/pancreas transplantation; patients from each transplant centre; patients with concurrent urinary tract infection at the time of biopsy/urine sampling; patients with concurrent CMV infection at the time of biopsy/urine sampling and patients with polyomavirus nephropathy.

Furthermore, the secondary analysis examines whether certain clinical, laboratory or pathological variables interfere with the mass spectrometric test. This facilitates the search for potential confounders and the definition of the limits of the MS test.

For the primary analysis no missing data due to drop-outs is expected because once a patient has consented to participate in the study the data for primary analysis is available. Drop-outs would only affect analysis of follow-up data (e.g. rejection events after the test).

Within the sensitivity analysis, the clinical course and subsequently performed biopsies (if available) will be additionally used for an assessment of the probable true disease state in case of a positive mass spectrometry result and a negative biopsy. Also, the results of the central read of the biopsy vs. the local pathological diagnosis will be used in the sensitivity analysis.

In a descriptive secondary analysis, the test reproducibility will be evaluated. Therefore, Krippendorff’s alpha will be calculated for the probe pairs (of 10 % of the patients).

### Ethical issues

All patients will be asked to consent into the collection and scientific analysis of urine samples and the clinical data in an anonymous fashion. Patients will be assured that the medical treatment will be not influenced by their decision to participate in the study.

As indicated biopsies are sought to be performed without much delay, the time to consider participation in the study might be rather short in some instances. Therefore, patients will be explicitly informed that they can withdraw their initially given consent.

### Financing

The trial is funded by the German research foundation (DFG) in the context of the clinical trials funding program (*Non-invasive diagnosis of acute rejection in renal transplant patients using mass spectrometry of urine samples - a multicentre diagnostic phase III trial*).

## Discussion

The perspective is that the MS test (respectively, a simplified test system derived from this method) could be used in the regular post-transplant surveillance for acute rejection, in place of the relatively insensitive procedure with periodic monitoring of the graft function by creatinine determinations. The MS test would serve as a decision help to decide whether an allograft biopsy is necessary in this setting to confirm the presence of rejection. In the long-term, the MS test might even replace some of the biopsies.

For the affected patients this would translate into a more timely detection of acute rejection and into fewer renal allograft biopsies, including avoidance of procedure-related complications. If the test proves to be successful in early and reliably identifying patients with a high probability of acute rejection, this would enable timely intervention and probably, prevention of renal tissue injury and of the deleterious long-term consequences of the rejection.

Assessing the potential economic impact is premature. In case, the test could be successfully applied in the routine post-transplant patient care, reduction in graft losses/increased graft survival has to be shown in this setting in a randomized diagnostic phase 4 study [6].

Furthermore, the proposed undertaking offers the unique perspective to use the well-characterized pool of collected and analysed samples in a later study to examine potentially interesting peptides, in an attempt to further delineate important pathways and processes of the rejection.

### Trial status

The study was reviewed by the leading Ethics Committee of the Medical School Hannover. In accordance with German law, this ethical approval is sufficient for all participating centres, because there is no intervention for the patients (the diagnostic test is an in-vitro test). The first patient was enrolled in October 2011. The clinical trial is ongoing and is expected to be finished in December 2015.

## Additional file

Additional file 1:
**Quality Assurance/Monitoring.** (DOC 31 kb)

## Abbreviations

GFR: Glomerular filtration rate
HLA: Human leukocyte antigen
MS: Mass spectrometry
SOP: Standard operating procedure
