# Supplementary material for: Non-invasive diagnosis of acute rejection in renal transplant patients using mass spectrometry of urine samples - a multicentre phase 3 diagnostic accuracy study
Source: BMC Nephrol. 2015 Sep 15;16:153. doi: 10.1186/s12882-015-0146-x (PMC4570292; doi:10.1186/s12882-015-0146-x)
Supplement: Additional file 1: — Quality Assurance/Monitoring. (DOC 31 kb) [file 12882_2015_146_MOESM1_ESM.doc]

**Supplement**

**Quality assurance / monitoring**

Quality of the study will be assured regarding the following issues:

1. All biopsies will be submitted to a repeat Central reading at the Department of Pathology of the Medical School Hannover. Cases with disparate diagnoses between the Central reading and the local pathologist will be re-evaluated and discussed between the two readers to agree on a final diagnosis. All biopsies will be assessed for adequacy of the material to make a reliable diagnosis according to the requirements set by the BANFF classification (13, 14). Adequacy of the biopsy will be recorded during the evaluation of the biopsy and considered in the data analysis.
2. The mass spectrometry test will be conducted according to internal SOP guidelines to assure equal handling and analysis of all samples. The SOP’s cover standardized methods for sample storage and preparation, CE-MS sample analysis, raw data processing by proprietary software (de-convolution of signals to single masses), for calibration and annotation of peptide lists, determination of membership probabilities according to characteristic peptide patterns as well as for storage of results in a database. Sample processing and measurement variability has been examined by (15). On the basis of these results (effects of temperature and time, sample stability with repeated freeze-thaw cycles) the CE-MS procedure has been adapted for routine laboratory analysis. Reproducibility of CE-MS measurements is ensured by periodic measurement of external polypeptide calibrants and by applying the following quality criteria to diagnostic testing: detection of a minimum of 800 peptides (sensitivity control) and identification of at least 130 of 200 internal marker peptides (specificity control) in a urine sample. Samples that do not fulfill these requirements are excluded from analysis and are again subjected to CE-MS.

Retain samples will be kept from all patients for repeat analysis of unclear results, cross-validation with other tests (e.g. mass spectrometry or other technologies from other research groups), and for peptide sequencing.

1. Collection, storage and shipping of the urine samples will be done according to a SOP which is circulated among all participating study centres. All samples will be labelled with unique identifiers to avoid confusion of samples. Adherence to the sample handling protocol will be recorded in the centres by including all pertinent information in the Case Report Form (date of sampling, time lag between collection and freezing, storage temperature, date and conditions of shipping).

From all urine collections a retain sample will be stored in aliquots for repeated analysis and for further examinations/tests.

1. All clinical and laboratory data collected at the study sites will be entered into an electronic Case Report Form (on the platform of SecuTrial® software). The software will be programmed to facilitate avoidance of missing and implausible entries. All entries will be monitored centrally by the Study Monitor at regular intervals and queries will be generated on unclear entries.

Three regular on-site monitoring visits are planned in each study centre at time points depending on the recruitment process.

1. Before final statistical analysis, the data will be validated by the Institute of Biostatistics of the Medical School Hannover, using pre-defined algorithms to detect implausible data and implausibly missing data and to identify outliers. Final statistical analysis will be done after clearance and correction of equivocal data.
2. Quality of essential parts of the study (process and results of the monitoring, data analysis, and pathology reading) will be evaluated by the DMSB.
